# Recent advances in PET/MR imaging for head and neck tumors: a systematic review of the last three years

**DOI:** 10.1007/s11547-025-02075-y

**Published:** 2025-08-28

**Authors:** Daniele Antonio Pizzuto, Minerva Becker, Patrick Veit-Haibach, Michael Messerli, Virginia Liberini, Antonio Giulio Gennari, Winnie Wing-Chuen Lam, Alexander Maurer, Tetsuro Sekine, Maria Picchio, Munenobu Nogami, Salvatore Annunziata, Ken Herrmann, Martin Huellner

**Affiliations:** 1 Department of Nuclear Medicine, University Hospital Zurich, University of Zurich, Zurich, Switzerland; 2 GSTeP Radiopharmacy, Dipartimento di Diagnostica per Immagini e Radioterapia Oncologica, Fondazione Policlinico Universitario A. Gemelli IRCCS, Rome, Italy; 3 Division of Radiology, Diagnostic Department, Geneva University Hospitals, University of Geneva, 1205 Geneva, Switzerland; 4 University Medical Imaging Toronto, Joint Department Medical Imaging, University Health Network, Sinai Health System, Women’s College Hospital, University of Toronto, Toronto, ON M5G 2N2 Canada; 5 Nuclear Medicine Unit, ASO S.Croce e Carle, 12100 Cuneo, Italy; 6 Department of Neurology, University Hospital Zurich, Zurich, Switzerland; 7 Department of Nuclear Medicine and Molecular Imaging, Singapore General Hospital, Singapore, 169608 Singapore; 8 Radiological Sciences Academic Clinical Program, Duke-NUS Medical School, Singapore, 169857 Singapore; 9 Department of Radiology, Nippon Medical School Musashi Kosugi Hospital, Kanagawa, Kawasaki Japan; 10 Vita-Salute San Raffaele University, Milan, Italy; Department of Nuclear Medicine, IRCCS Ospedale San Raffaele, Milan, Italy; 11 Department of Radiology, Kobe University Hospital, Kobe, Japan; 12 Biomedical Imaging Research Center, University of Fukui, Fukui, Japan; 13 Department of Nuclear Medicine, West German Cancer Center, University Hospital Essen, University of Duisburg-Essen, Essen, Germany

**Keywords:** PET/MR, Head and neck, Hybrid imaging, Tumor metabolism, Prognosis

## Abstract

**Purpose:**

This systematic review addresses the clinical relevance of PET/MR in patients with head and neck (HN) tumors, highlighting studies conducted over the last three years to provide an updated perspective on integrating a hybrid PET/MR scan into different clinical scenarios.

**Methods:**

We employed a search algorithm, combining terms (“PET/MR” OR (“PET” AND (“MR” OR “MRI”)) OR “PET/MRI” OR “PET-MR” OR “PET-MRI” OR (“PET” AND “magnetic”)) AND (“head” AND “neck”). Studies written in English and published throughout 2021, 2022 and 2023 up to November 15th were considered if were focused on: suspected HN tumors; confirmed HN tumors before surgery/radiotherapy/chemotherapy; HN tumor recurrence or therapy response assessment. Reviews, editorials or letters, case report/series or any other original unrelated studies to the topics were excluded.

**Results:**

Twenty out of 169 studies were deemed eligible. The HN tumor cohorts included sinonasal tumors, nasopharyngeal carcinoma and tumors in the oropharynx, oral cavity, hypopharynx and larynx, mostly of squamous cell carcinoma histology. Sixteen out of 20 articles focused on initial staging, including prognostic information before primary treatment, whereas 4/20 articles explored the clinical significance of PET/MR in restaging settings or other clinical purposes.

**Conclusion:**

This review consolidates previous findings by showing the relationship between morphology, metabolism, cellularity, and perfusion in HN tumors. Metrics provided by PET/MR are able to predict the histologic grade of HN tumors, EGFR status and patient outcome. PET/MR demonstrates high diagnostic performance for detecting locoregional tumor recurrence, distant metastases and second primary cancers.

## Introduction

Hybrid positron emission tomography combined with magnetic resonance (PET/MR) imaging integrates the high soft-tissue contrast of MRI with the high sensitivity and functional information of PET in a single acquisition. This technique has been explored for its potential advantages in oncologic imaging, particularly in complex anatomical regions, such as the head and neck (HN).

In HN tumors, PET/MR may provide improved anatomical detail and functional characterization, supporting precise delineation for staging and treatment planning. Several studies have compared PET/CT and PET/MR in clinical settings [1, 2]. However, most of them are retrospective studies with heterogeneous tumor types, sites, and indications, limiting the generalizability of results. Prospective investigations specifically targeting squamous cell carcinoma (HNSCC) remain limited [3, 4].

To address these limitations and provide a timely update, we conducted a systematic review restricted to studies published in the past 3 years—since the last comprehensive review on this topic dates back to 2020 [5]. A recent meta-analysis by Al-Ibraheem et al. assessed the diagnostic performance of [^18F]FDG PET/MRI in HNSCC, providing pooled sensitivity and specificity estimates [6]. However, their focus remained on imaging metrics, without addressing how PET/MR is used across different clinical settings or its impact on therapeutic decision-making. Our review complements and updates previous work by examining the most recent literature with specific attention to clinical integration and potential influence on patient management.

To our knowledge, no recent review has systematically assessed the evolving clinical role of integrated PET/MR in head and neck tumors, specifically with regard to focus on staging, restaging, prognostication, and treatment response in studies published over the last three years.

Therefore, this review aims to address the diagnostic and prognostic value of integrated PET/MR in the evaluation of head and neck tumors, and how it impacts clinical decision-making in staging, restaging, and treatment response assessment.

## Materials and methods

### Search strategy

Two researchers (D.A.P and M.W.H) conducted a comprehensive literature review using the PubMed/MEDLINE, Embase databases, and the Cochrane Library database to identify articles on the clinical utilization of PET/MR in assessing HN tumors. The following terms were applied in various combinations:

(“PET/MR” OR (“PET” AND (“MR” OR “MRI”)) OR “PET/MRI” OR “PET-MR” OR (“PET” AND “magnetic”)) AND (“head” AND “neck”). The search covered studies published from 2021, to 2023 November 15th. The full search strategy is available from the authors upon reasonable request.

Eligibility Criteria.

Studies were included if they:

- Reported on human subjects with suspected or confirmed head and neck tumors
- Used integrated PET/MR for primary staging, restaging, recurrence detection, or response assessment
- Provided clinical or diagnostic data relevant to PET/MR performance or interpretation

Exclusion criteria:

- Reviews, editorials, letters, case reports, case series
- Non-oncological applications or unrelated anatomical regions
- Technical/methodological papers focusing on image acquisition or attenuation correction

Original articles that included both PET/CT and PET/MR were considered if PET/MR results could be separately extracted.

#### Study selection

After duplicate removal, titles and abstracts were independently screened by two reviewers (D.A.P. and M.W.H.) based on the eligibility criteria. Full texts of potentially eligible articles were retrieved and reviewed. Any disagreement was resolved through discussion and consensus. References of the included studies were also manually screened to identify additional relevant articles.

#### Data extraction

For each included study, data were extracted independently by the above-mentioned reviewers, by using a predefined extraction template. The following variables were systematically recorded:

- *Study characteristics*: first author, year of publication, country, study design (prospective vs. retrospective)
- *Patient population*: sample size, age, sex distribution, tumor site, histological subtype
- *Clinical context*: indication for PET/MR (initial staging, restaging, recurrence detection, or therapy response)
- *Imaging details*: radiotracer used, PET and MR parameters (e.g., SUVmax, ADCmean, Ktrans), sequences acquired
- *Reference standard*: histopathology, imaging follow-up, multidisciplinary decision
- *Comparators*: PET/CT, MRI alone, or other modalities (if reported)
- *Outcomes*: diagnostic performance (sensitivity, specificity, accuracy), prognostic value (e.g., survival correlations, prediction of response)

Discrepancies in data extraction were resolved by consensus. Diagnostic performance data (sensitivity, specificity, and AUC) were extracted where available and summarized in two ways: a summary table grouping studies by clinical application, and a visual representation through descriptive forest plots including studies with at least two diagnostic metrics.

This review followed PRISMA principles to enhance transparency and reproducibility of methods [7].

## Results

**Fig. 1:**
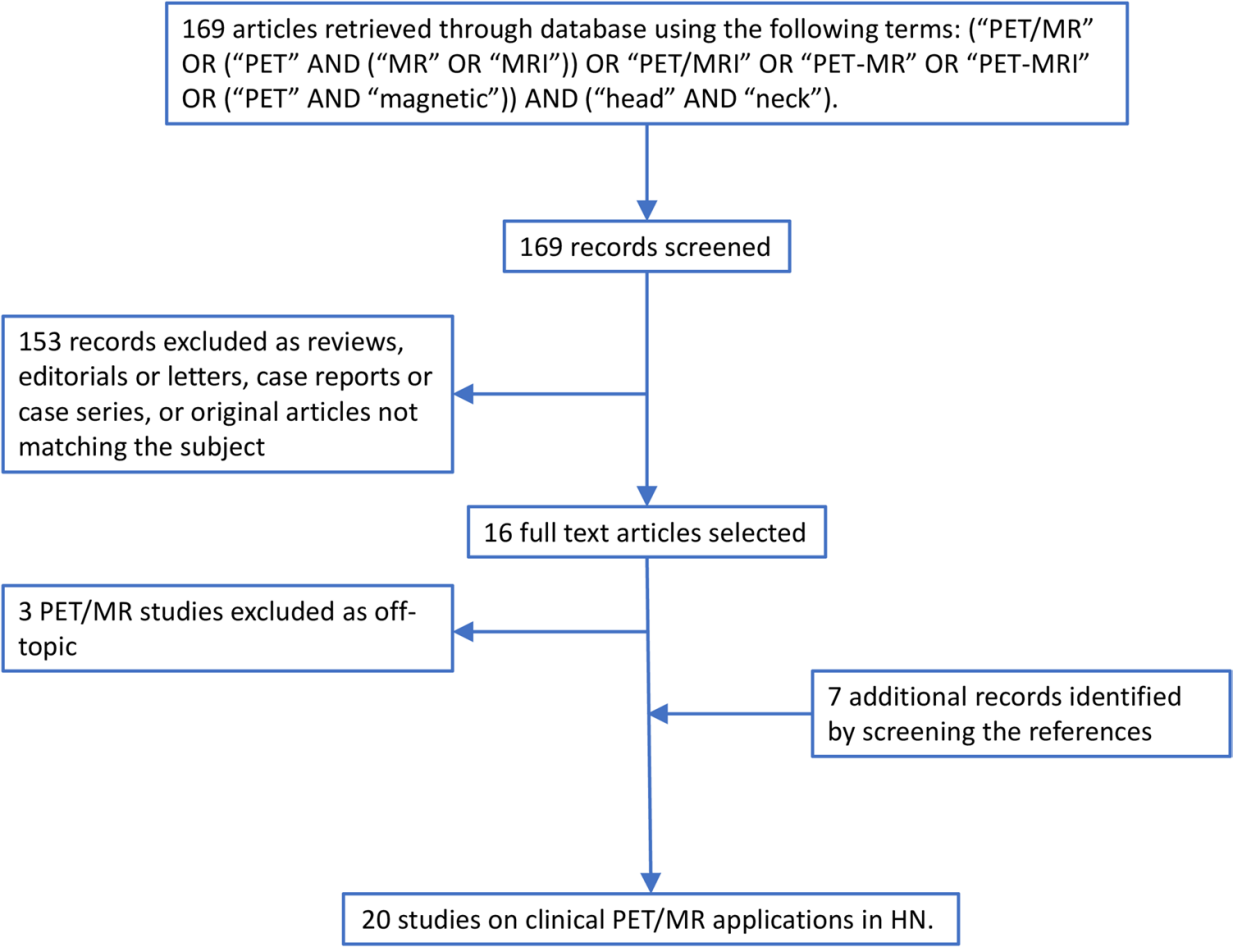
The stepwise article selection process

A total of 169 articles were initially screened, with 153 records excluded as they were reviews, editorials or letters, case reports/series or original articles with unrelated topic. Seven more records were incorporated after screening the references of the included articles, resulting in 23 full-text articles. Three out of 23 articles were excluded, as addressed novel technology (including deep learning) concerning attenuation correction, rather than clinical application of PET/MR scans in HNSCC patients. Twenty studies (15 retrospective and 5 prospective) were deemed eligible. A flowchart illustrates the selection process (Fig. 1). The HN tumor cohorts included sinonasal tumors (SN), melanoma or adenocarcinoma, nasopharyngeal carcinoma (NPC, including adenocarcinoma), and tumors located in the oropharynx (OP), oral cavity, hypopharynx (HP) and larynx, mostly SCC. Table 1 lists included articles and patient data (e.g., sex, age, primary disease site). Table 2 outlines the specific focus of included article, such as the study objective, the clinical purpose of PET/MR, the type of image analysis, the standard of reference and other possible comparators.

**Table 1:** Overview of studies covered in this review (setup and specifications)

Authors	Year	Country of origin	Study design	Number of patients (PET/MR exams)	Patient age, mean ± SD or median (range/IQR)	Sex (% male)	Primary tumor location
Freihat et al. [18]	2021	Hungary	Retrospective	33 (33)	61 ± 0.7	70	Oropharynx
Crimì et al. [3]	2021	Italy	Retrospective	26 (26)	58 (SD NR)	69	Oral cavity, oropharynx, sinonasal
Pace et al. [19]	2021	Italy	Retrospective	45 (45)	60 ± 11	84	Oropharynx, hypopharynx
Cao et al. [4]	2021	China	Retrospective	331 (331)	51 (13–81)	77	Nasopharynx
Piao et al. [22]	2021	China	Retrospective	60 (60)	51 (26–73)	75	Nasopharynx
Patel et al. [23]	2021	USA	Retrospective	37 (46)	NR	NR	Sinonasal, nasopharynx
Valdec et al. [25]	2022	Switzerland	Retrospective	7 (7)	72 (23–82)	86	Head and neck
Murtojarvi et al. [24]	2022	Finland	Retrospective	52 (52)	64 ± 11	69	Nasopharynx, oral cavity, oropharynx, larynx, hypopharynx, unknown primary
Meng et al. [8]	2022	China	Retrospective	21 (21)	55 ± 6	100	Hypopharynx
Katirtzidou et al. [9]	2022	Switzerland	Prospective	103 (103)	61 ± 12	85	Oral cavity, oropharynx, hypopharynx, larynx
Huang et al. [20]	2022	China	Prospective	21 (21)	55 ± 6.3	100	Hypopharynx
Freihat et al. [10]	2022	Hungary	Retrospective	71 (71)	61.6 ± 0.8	69	Pharynx, larynx, oral cavity
Flygare et al. [11]	2022	Sweden	Retrospective	40 (40)	64 (SD NR)	80	Oropharynx
Chan et al. [21]	2022	Taiwan	Prospective	144 (144)	60 ± 10	93	Oropharynx, hypopharynx
Wongsa et al. [12]	2023	Thailand	Prospective	11 (11)	60 (48–78)	73	Salivary glands, nasopharynx, oral cavity
Terzidis et al. [13]	2023	Denmark	Prospective	13 (13)	NR	NR	Oral cavity, oropharynx, hypopharynx
Fang et al. [14]	2023	China	Retrospective	58 (58)	49 (15–69)	79	Nasopharynx
Chen et al. [15]	2023	China	Retrospective	21 (21)	NR	85	Oral cavity, hypopharynx, larynx
Cebeci et al. [16]	2023	Turkey	Retrospective	44 (44)	66 (19–85)	84	Sinonasal, hypopharynx, larynx, salivary glands
Cao et al. [17]	2023	China	Retrospective	343 (343)	51 (13–81)	77	Nasopharynx

*IQR* interquartile range, *MR* magnetic resonance, *NR* not reported, *PET* positron emission tomography, *SD* standard deviation

**Table 2:** Overview of studies covered in this review (purpose and main results)

Clinical Application	Authors	Year	Purpose	Main results	Image analysis	Standard of reference	Comparators
Primary tumor assessement	Freihat et al. [18]	2021	Prediction of HPV status and local tumor response to curative chemoradiotherapy	ADC_mean_ predicts HPV status. ADC_mean_, MTV and TLG predict therapy response	Visual and semiquantitative	Biopsy and clinical follow-up (duration not mentioned)	NR
Lymph node staging	Crimì et al. [3]	2021	Diagnostic accuracy in nodal staging	High diagnostic accuracy, NPV and PPV of PET/MR using PET SUV_max_ cutoff of 5.7	Visual and semiquantitative	Histopathology (neck dissection)	CE neck MR
Prognostication	Pace et al. [19]	2021	Prognostic value in advanced OHPSCC	SUL_peak_ and SUL_peak_/ADC_mean_ of primary tumor as predictors of OS (univariate analysis). SUL_peak_ as independent predictor of OS (multivariate analysis)	Semiquantitative	Survival data (mean follow-up = 31 months)	NA
Primary tumor assessement	Cao et al. [4]	2021	Evaluation of locoregional extension pattern of NPC and CTV delineation	NPC spreads in a systematic, stepwise fashion from proximal to distal and disease skipping is unusual	Visual	none	NA
Distant metastases	Piao et al. [22]	2021	Detection and staging of recurrent/metastatic NPC	Diagnostic accuracy of 88.3% for detecting recurrent/metastatic NPC	Visual	Histopathology/at least 6 months imaging follow-up	NA
Post-treatment surveillance	Patel et al. [23]	2021	prediction of local recurrence and or progression after treatment using NI-RADS scores adapted for PET/MR	High diagnostic accuracy for detecting treatment failure with PET/MR NI-RADS scores (AUC range, 0.86–0.99)	Visual	Histopathology (biopsy)/clinical or imaging follow-up (median = 8.9 months after treatment)	CE neck MR
Other clinical purposes	Valdec et al. [25]	2022	Dental focus assessment	No association between signs of inflammation on dental radiographs, clinical percussion data and PET/MR findings	Visual and semiquantitative	Clinical outcome (percussion sensitivity)	OPT, dental X-rays
Post-treatment surveillance	Murtojarvi et al. [24]	2022	Diagnostic performance in detecting local Recurrence	AUC for PET/MR and PET/CT on patient-based analysis was 0.997 and 0.890, respectively	Visual and semiquantitative	Histopathology, imaging follow-up	PET/CT
Primary tumor assessment	Meng et al. [8]	2022	Prediction of histologic grade of HSCC using a combined model of SUV_mean_, ADC and Ki	AUC of combined model was 0.94 with sensitivity of 90% and specificity of 81.8%	Quantitative	Histopathology (biopsy)	NA
Distant metastases	Katirtzidou et al. [9]	2022	Diagnostic performance of PET/MR for detection of distant metastases and second primary tumors	Diagnostic performance of PET/MR and PET/CT was similar with AUCs of PET/MR versus PET/CT ≥ 0.944 in the per patient, per examination and per lesion analysis for both techniques	Visual and semiquantitative	Histopathology (biopsy) and/or follow-up > 2 years	PET/CT
Prognostication	Huang et al. [20]	2022	Prognostic value of PET/MR in HPSCC	MTV and TLG as independent predictors of PFS and OS, respectively	Semiquantitative	Clinical and imaging follow-up (median = 20.3 months)	NA
Primary tumor assessment	Freihat et al. [10]	2022	Correlation between tumor metabolism and cellularity, correlation with N stage	SUV_max_ correlates with N. stage. TLG of primary tumor correlated with T stage and N stage. ADC correlates with degree of tumor differentiation	Semiquantitative and quantitative	Histopathology (biopsy) of the primary tumor	NA
Primary tumor assessment	Flygare et al. [11]	2022	Evaluation of T and N staging of OPSCC with PET/MR and PET/CT and evaluation of reader agreement	Similar PET/MR and PET/CT sensitivity (≥ 0.97 and 1.00, respectively) and similar NPV (≥ 0.95 and 1.00, respectively). Interobserver agreement higher for PET/MR than for PET/CT	Visual	Histopathology (biopsy) of primary tumor. TN stage defined at the multidisciplinary tumor board	PET/CT
Prognostication	Chan et al. [21]	2022	Prognostic value before chemotherapy or radiotherapy	MTV, K rate constant (i.e., K_trans_) as independent predictor of OS and RFS	Semiquantitative and quantitative	Clinical follow-up	NA
Primary tumor assessment	Wongsa et al. [12]	2022	PET and MR metrics correlation	Inverse correlation between SUV and ADC values	Semiquantitative	NA	Phantom
Primary tumor assessment	Terzidis et al. [13]	2023	Correlation between tumor volume on imaging and pathology	Mean GTV_ONCO_ was larger than GTV defined by PET or MR. Mean mismatch of GTV_PATO_ compared to GTV_PET_, GTV_MR_ and GTV_ONCO_ was 31.9%, 54.5% and 27.9%, respectively	Semiquantitative	Histopathology of resected surgical specimen	NA
Distant metastases	Fang et al. [14]	2023	Detection and assessment of NPC bone metastases	PET/MR more sensitive than PBS in lesion-based analysis (100.0% vs. 50%, respectively); similar sensitivity in the patient-by-patient analysis	Visual	Histopathology or clinical follow-up	PBS
Primary tumor assessment	Chen et al. [15]	2023	Diagnostic value and Correlation of PET/MR metrics with EGFR	SUV_mean_ significantly different between high-grade and low-grade EGFR expression subgroups	Semiquantitative	Histopathology (biopsy)	NA
Lymph node assessment	Cebeci et al. [16]	2023	Efficacy of PET/MR in determining neck nodal status in HNSCC patients with cN0 necks	PET/MR more effective than MR or PET alone in distinguishing pathological N0 and N + patients (90.9% acc) in the patient-by-patient analysis	Visual	Histopathology (neck dissection)	MR and PET alone
Distant metastases	Cao et al. [17]	2023	cost-effectiveness and clinical value of a metastatic staging PET/MR	More patients with FP results in CWU group than in PET/MR group	Visual	Imaging follow-up/clinical follow-up	Conventional work-up

*Acc* accuracy, *ADCmean* mean apparent diffusion coefficient, *ARI*, additional radiological information, *AUC* area under the curves, *CE* contrast-enhanced, *ChT* chemotherapy, *CT* computed tomography, *CTV* clinical tumor volume, *CWU* clinical work-up DCE dynamic contrast-enhanced, *ENE* extranodal extension, *FP* false positive, *GTVMR* gross tumor volume defined by MR, *GTVPET* gross tumor volume defined by PET, *GTVONCO* gross tumor volume defined by clinical information, *GTVPATO* pathological tumor delineation, *HNC* head and neck cancer, *HNSCC* head and neck squamous cell carcinoma, *HSCC* hypopharyngeal squamous cell carcinoma, HPV human papilloma virus, *IVIM* intravoxel incoherent motion, Ktrans volume transfer constant, MR magnetic resonance, *MTV* metabolic tumor volume, *NA* not applicable, *NI-RADS* neck imaging reporting and data system, *NPC* nasopharyngeal carcinoma, *NPV* negative predictive value,, *NR* not reported, *OHSCC* oropharyngeal and hypopharyngeal squamous cell carcinoma, *OPSCC* oropharyngeal squamous cell carcinoma, *OPT* orthopantomogram, *OS* overall survival, *PBS* planar bone scan, *PET* positron emission tomography, *PFS* progression free survival, *PPV* positive predictive value, *RT* radiotherapy, *RFS*, recurrence-free survival, *ROC* receiver operating characteristics, *SULpeak* peak standardized uptake value corrected for lean body mass, *SUVmax* maximum standardized uptake value, *SUVmean* mean standardized uptake value, *TLG* total lesion glycolysis

### PET/MR in primary HN cancers

Sixteen out of the 20 studies focused on in newly diagnosed HN tumor patients, i.e., initial staging, evaluating tumor spread, predicting the histologic grade of SCC according to quantitative metrics and acquiring prognostic information before primary treatment, typically surgery and/or (chemo)radiotherapy [3, 4, 8–21] (Table 1, Table 2).

#### Primary tumor assessment

Cao et al. retrospectively evaluated the patterns of local tumor extent and the potential nodal involvement using PET/MR in a newly diagnosed NPC patients (*n* = 331). Regions were classified as high-risk (e.g., foramen lacerum, clivus), medium risk (e.g., cavernous sinus, parotid gland) and low risk (e.g., orbit, hypopharynx) for neoplastic invasion. The authors found that NPC spreads systematically and stepwise from proximal to distal with unusual primary disease skipping. Using PET/MR, the rates of metastatic lymph nodes decreased from the upper to the lower neck, and skip nodal metastases were present in about 2% of patients [4]. PET/MR could be beneficial for gross tumor volume (GTV) and clinical target tumor volume (CTV) delineation considering both morphological and functional aspects; GTV corresponds to the measurable, segmented tumor, whereas CTV corresponds to GTV with an additional margin, reflecting potential microscopic disease. This is supported by Terzidis et al., who compared integrated [^18^F]F-FDG PET/MR-based tumor volumes and pathological tumor volumes from surgical specimens in 13 HNSCC patients with various primary tumor sites. They evaluated the overlap/mismatch between the GTV of the resected tumor specimen (GTV_patho_) and the GTVs from PET segmentation (GTV_PET_), MRI segmentation (GTV_MRI_) and segmentation based on combined PET/MR data and clinical information (GTV_onco_). Furthermore, the authors analyzed the addition of 5 mm margin before re-assessing overlap/mismatch. The mean mismatch, i.e., the percentage of GTV_patho_ not encompassed in the GTV_onco_ was 27.9%, but the addition of a 5 mm margin to GTV_onco_ (CTV) resolved such discrepancies in 11/13 (84.6%) cases [13].

Meng et al. investigated whether [^18^F]F-FDG-PET quantitative parameters, diffusion-weighted imaging (DWI), and dynamic contrast-enhanced magnetic resonance imaging (DCE-MR) could predict the histologic grade in HPSCC patients (*n* = 21) undergoing PET/MR primary staging with endoscopic biopsy as gold standard. Significant differences in mean standardized uptake value (SUV_mean_), mean apparent diffusion coefficient (ADC_mean_) and volume transfer constant (K_trans_) were observed between subgroups of HPSCC patients categorized as poorly or moderately differentiated. The combination of SUV_mean_, ADC_mean_ and K_trans_ yielded a sensitivity of 90% and a specificity of 81.8% for estimating the histologic grade of HPSCC [8].

Freihat et al. investigated the disease aggressiveness, by PET and MR simultaneously in 71 patients with recently diagnosed HN tumors. Biopsy-based histology was the gold standard. Their analysis revealed no correlation between the information obtained by DWI and [^18^F]F-FDG-PET information, concluding that both imaging techniques could play a complementary role in the diagnosis and assessment of HN tumors [10].

Flygare et al. compared the diagnostic performance of PET/MR and PET/CT for assessing the primary tumor in 36 recently diagnosed OPSCC patients. No significant differences in tumor staging or tumor size between the two modalities were found. The gold standard for the TN stage was the final decision of a multidisciplinary tumor board [11].

Wongsa et al. correlated PET-SUV values and ADC from DWI sequences of primary tumor in 11 patients undergoing PET/MR for staging. The authors observed a significant inverse correlation between SUV_max_ and ADC_mean_ (*r* = − 0.75, *p* = 0.01), suggesting a combination of both to predict treatment response [12].

Chen et al. investigated the potential added value of combining semiquantitative PET, DWI and DCE parameters (SUVvalues, ADC_mean_ and K_trans_) to predict the epidermal growth factor receptor (EGFR) status in 21 primary HNSCC cases. Both PET and MR-derived diffusion and perfusion parameters showed a high accuracy (Area Under the Curve, AUC: 0.93), a sensitivity of 1.00 and a specificity of 0.86 [15].

Freihat et al. retrospectively compared the PET/MR parameters (SUV_max_, Total Lesion Glycolysis (TLG) Metabolic Tumor Volume (MTV), ADC) to clinicopathological characteristics, like T and N classification, tumor grade, HPV status and treatment response in 33 OPSCC patients. ADC_mean_ was significantly lower in the subgroup of HPV-positive patients; [^18^F]F-FDG-PET parameters (MTV, TLG and SUV_max_) uncorrelated to HPV status [18].

#### Lymph node assessment

Crimì et al. compared PET/MR and contrast-enhanced MR (ceMR) for detecting lymph node metastases in 26 HNSCC patients, with histopathology as gold standard. The authors reported a sensitivity of 74.3% and specificity of 97.6% for PET using MR for anatomic localization, while a sensitivity of 60.0% and specificity of 99.4% for ceMR using both dimensional and morphological criteria. With a SUV_max_ cutoff of 5.7, PET/MR achieved accuracy, sensitivity, specificity, for the detection of lymph node metastases of 98.2%, 57.1%, 99.8%, respectively. With an SUVmax cutoff of 5.7, PET/MR achieved an AUC of 0.982, which was significantly higher than the AUC of ceMRI (0.868) using dimensional and morphologic criteria (*p* < 0.05)[3].

Cebeci et al. proposed a superior diagnostic performance of PET/MR compared to PET and/or MR alone in detecting occult lymph node metastases in 44 HPV-negative HNSCC patients with clinically negative necks. PET/MR was conducted before neck dissection, serving as standard of reference. The patient-by-patient analysis included 38 patients with pathological N0 (pN0) necks and 6 patients with pathological N + (pN +) necks. According to histopathological results, MRI had 50.0% sensitivity, 89.5% specificity, and 84.0% accuracy, while PET had 83.3% sensitivity, 68.4% specificity, and 70.4% accuracy. PET/MRI was more successful in distinguishing pN0 and pN + cases (83.3% sensitivity, 92.1% specificity, negative predictive value (NPV) of 97.2%, positive predictive value (PPV) of 62.5%, and 90.9% accuracy) [16].

**Fig. 2:**
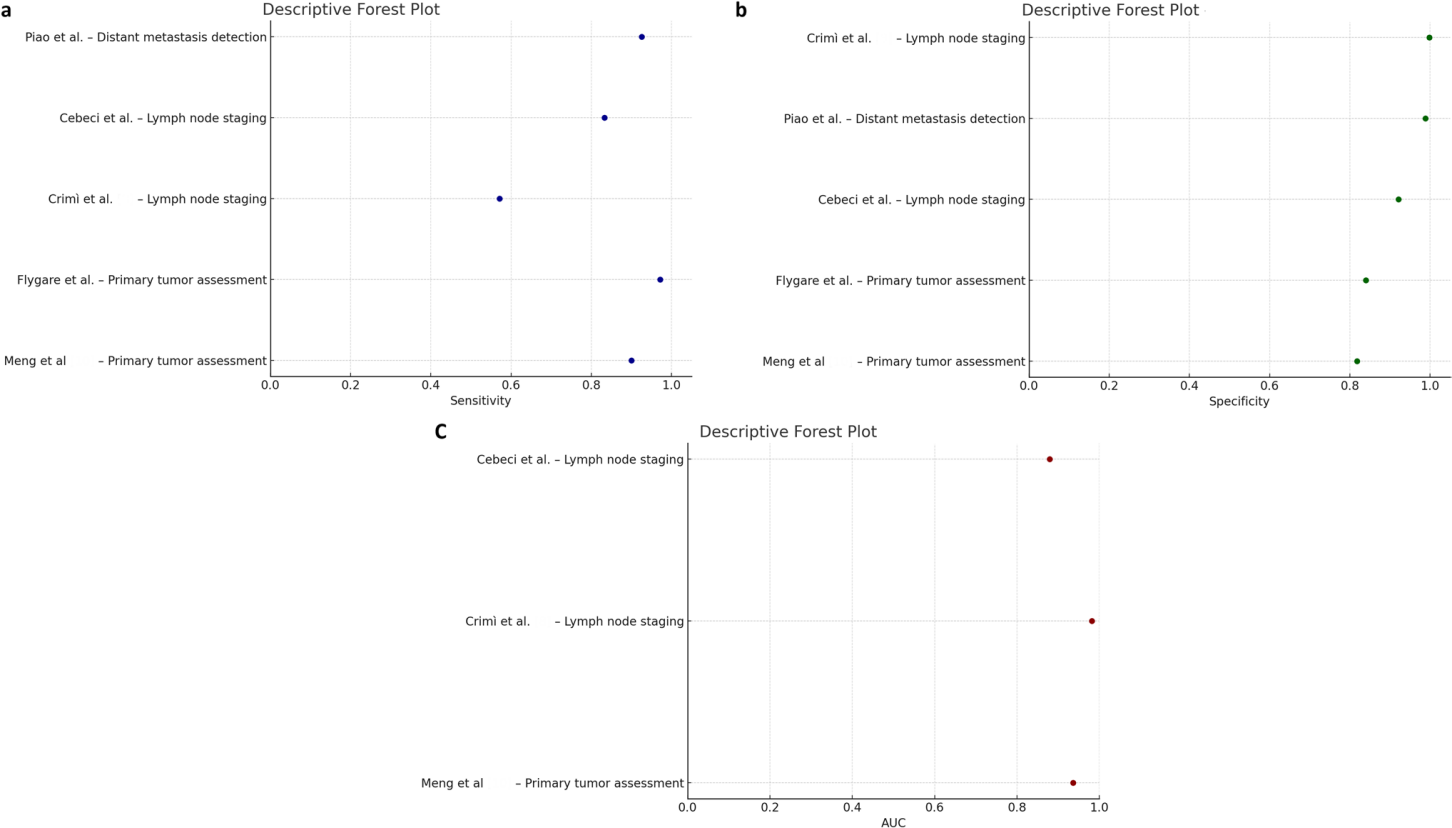
Descriptive forest plots summarizing the diagnostic performance of integrated [^1^⁸F]FDG PET/MR across selected clinical applications. Separate panels display reported values of sensitivity (**a**), specificity (**b**), and area under the ROC curve (AUC) (**c**) from eligible studies that included at least two of these diagnostic metrics. Each point represents a single study and clinical setting, plotted according to the reported value. No pooling or meta-analytic weighting was applied. Studies are grouped by clinical application and labeled by first author. Data were extracted directly from the original articles included in this systematic review

Freihat et al. investigated the predictive value of [18F]F-FDG PET parameters (SUV_max_, TLG and MTV) and ADC_mean_ values of PET/MR for the N stage in 71 HNSCC patients. SUV_max_ and TLG correlated to N stages, unlike MTV and ADC_mean_ [10]. Tumor size and N stage were independent factors influencing SUV_max_. To visually summarize the heterogeneity in diagnostic performance across studies, three descriptive forest plots were generated, illustrating the reported sensitivity, specificity, and AUC across clinical key applications (Fig. 2).

#### Distant metastases

Katirtzidou et al. compared the diagnostic performance of PET/MR and PET/CT for detecting distant metastases and second primary cancers in a prospective head-to-head study including 82 HNSCC patients (38 patients imaged for primary staging, 41 for suspected locoregional HNSCC recurrence and 3 for neck node metastases from an unknown primary HNSCC) [9]. The PET/MR protocol included whole-body contrast-enhanced Dixon-type sequences, no DWI. The standard of reference was histology of biopsy in 24 patients and imaging follow-up of more than 2 years in the remaining 58 patients. Distant metastases were present in 14/103 (13.6%) examinations in 7/82 (8.5%) patients; distant second primary cancers occurred in 9/103 (9%) patients. Most metastases were in the lung, bone, and mediastinal/hilar lymph nodes. PET/MR and PET/CT exhibited high and similar diagnostic performance with AUCs of PET/MR and PET/CT per-patient, per-examination and per-lesion of 0.947 vs. 0.975, 0.965 vs. 0.968 and 0.957 vs. 0.944, respectively (*p* > 0.05). The authors concluded that the diagnostic performance of both hybrid imaging tools could be similar, owing to the FDG avidity of distant metastases and second primary cancers in most of the cases.

Piao et al. evaluated the diagnostic performance of FDG-PET/MR for detecting distant metastases in recurrent or metastatic 60 NPC patients [22]. With histology or follow-up as reference, the sensitivity, specificity, PPV, NPV, and accuracy in detecting distant metastases were 92.5%, 98.8%, 97.4%, 96.3%, and 96.7%, respectively [22].

Fang et al. investigated the role of [^18^F]F-FDG PET/MR in detecting bone metastases, comparing its performance with planar bone scintigraphy (PBS) in 58 NPC patients [14]. The standard of reference was either lesion histology or follow-up > 6 months. PET/MR demonstrated greater sensitivity than PBS in lesion-based analysis (sensitivity 100.0% vs. 50.0%; *p* < 0.001).

Cao et al. conducted a cost-effectiveness analysis of [^18^F]F-FDG PET/MR in 343 NPC patients compared to standard clinical work-up (CWU). The primary endpoint was the false positive rate (FP) derived from [^18^F]F-FDG PET/MR, compared to FP from CWU imaging in 677 NPC patients. The FP rate was significantly lower with PET/MR than with CWU. The incremental cost-effectiveness ratio (ICER) was $54 for each percent of patients avoiding FP findings, indicating that PET/MR was cost-effective for initial staging [17].

#### Prognostication

Four studies investigated the prognostic role of PET/MR, by assessing PET and MR parameters from primary HN tumors, by examining associations between imaging-derived metabolic and functional parameters and survival outcomes, such as OS and PFS [18–21].

Freihat et al. retrospectively assessed the prognostic value of PET/MR, in predicting therapy response in 33 OPSCC patients [18]. ADC_mean_, TLG and MTV were predictive parameters of treatment response (*p* = 0.017, *p* = 0.013, *p* = 0.014, respectively); SUV_max_ was similar in complete (CR) and non-complete response (NCR) patients’ subgroups.

Pace et al. evaluated the prognostic significance of [^18^F]F-FDG-PET/MR in 45 patients with locally advanced OPSCC and HPSCC before chemoradiotherapy. The peak standardized uptake value corrected for lean body mass of the primary lesion (SUL_peak_) from PET images and SUL_peak_/ADC_mean_ were significant predictors of OS at univariate analysis. SUL_peak_ was further independent predictor at multivariate analysis [19].

Huang et al. retrospectively assessed the prognostic role of pre-treatment PET/MR in 21 HPSCC patients, analyzing progression-free survival (PFS) and OS [20]. At multivariate analysis, MTV and TLG were found independent prognostic factors of PFS and OS, respectively.

Chan et al. prospectively explored the prognostic role of [^18^F]F-FDG-PET/MR acquiring intravoxel incoherent motion (IVIM) and DCE MRI to assess survival in 144 OPSCC and HPSCC patients treated by chemoradiotherapy. The authors found that T classification, MTV and true water diffusion (D*) were independent risk factors for OS; T classification, N classification and K_trans_ predicted recurrence-free survival (RFS) [21].

All four studies applied PET/MR-derived quantitative parameters in pre-treatment settings and assessed their association with OS and/or PFS, using univariate and multivariate models.

### PET/MR in post-treatment surveillance

Three studies explored clinical applications of PET/MR in restaging HNSCC patients [22–24]. Piao et al. evaluated the accuracy of [^18^F]F-FDG-PET/MR in detecting local and lymph node recurrence in NPC patients [22]. The gold standard for locoregional recurrence was either the biopsy-based histology or imaging follow-up of at least 6 months. The sensitivity, specificity, PPV, NPV and accuracy of PET/MR in detecting local recurrence were 100%, 91.4%, 89.3%, 100% and 95.0%, and for recurrent lymph node metastases were 100%, 85.3%, 83.9%, 100% and 91.7%, respectively [22].

Patel et al. retrospectively assessed the performance of a post-treatment contrast-enhanced PET/MR in detecting treatment failure in HN tumor patients [23]. A total of 46 post-treatment PET/MR imaging studies in 37 patients were analyzed, with sinonasal tract and nasopharynx like common primary locations, and SCC like prevalent histotype (17/37, 45.9%). Treatment outcome was defined by follow-up. The authors utilized the Neck Imaging Reporting and Data System (NI-RADS) scores, ranging from 1 to 3–1 no suspicious, 2 low suspicion, and 3 high suspicion of recurrence, to evaluate how effective was the post-treatment PET/MR imaging to predict treatment failure in 92 primary and neck sites. NI-RADS PET/MR imaging scores strongly correlated to treatment failure for the primary site, neck lymph nodes, and combined sites (based on univariate association analysis; all *p* < 0.001). PET/MR imaging scores accurately predicted treatment failure (AUC 0.864–0.987, *p* < 0.001).

Murtojarvi et al. investigated the potential benefit of PET/MR compared to PET/CT in post-treatment follow-up imaging of HNSCC patients. Histopathological sampling or imaging follow-up of at least 2 years after treatment served as standard of reference. The recurrence occurred after 12 months was presumed independent of PET findings. In the patient-based analysis, PET/MR demonstrated higher sensitivity (1.00 vs. 0.67) and superior NPV (1.00 vs. 0.87) in detecting locoregional recurrence than PET/CT. In the patient-based and lesion-based analysis, the AUCs of PET/MR were superior to those of PET/CT (0.997 and 0.989 vs. 0.890 and 0.899), suggesting a potential advantage of PET/MR in post-treatment surveillance [24]. Reported sensitivity, specificity, and AUC grouped by clinical application are summarized in Table 3.

**Table 3:** Summary of diagnostic performance metrics reported across included PET/MR studies, grouped by clinical application

Clinical application	Authors	Year	Sensitivity	Specificity	AUC	Notes
Distant metastasis detection	Piao et al. [22]	2021	92.5	98.8	NR	NPC patients; post-treatment PET/MR; reference = histology or imaging follow-up
Distant metastasis detection	Katirtzidou et al. [9]	2022	NR	NR	0.947–0.965	AUC range for PET/MR (per-patient and per-exam); comparison with PET/CT; reference = histology or 2-year follow-up
Lymph node staging	Crimì et al. [3]	2021	57.1	99.8	0.982	PET/MR using SUVmax cutoff 5.7; histology as gold standard
Lymph node staging	Cebeci et al. [16]	2023	83.3	92.1	0.879	HPV-negative, N0 neck: surgical histology as reference; PET/MR pre-operative
Post-treatment surveillance	Patel et al. [23]	2021	NR	NR	0.864–0.987	NI-RADS PET/MR scores; AUC range for primary site and neck combined
Primary tumor assessment	Meng et al. [8]	2022	90.0	81.8	0.936	PET/MR multiparametric model (SUVmean + ADCmean + Ktrans); outcome = histologic grade
Primary tumor assessment	Flygare et al. [11]	2021	97.1	84.0	NR	Reader 1 (experienced); gold standard = tumor board consensus; PET/MR vs. PET/CT

Sensitivity, specificity, and AUC values are reported where available

*AUC* Area under the curve, *ADCmean* mean Apparent diffusion coefficient, *HPV* human papilloma virus, *ktrans* volume transfer constant, *NI—RADS* neck imaging reporting and data system, *NPC* nasopharyngeal carcinoma, *N*R not reported, *PET/CT* Positron emission tomography/computed tomography, *PET/MR* Positron emission tomography/magnetic resonance, *PFS* Progression-free survival, *SUVmax* maximum Standardized uptake value, *SUVmean* mean Standardized uptake value

### Other clinical purposes

Valdec et al. explored the feasibility of PET/MR for assessing inflammatory dental foci in 7 HN tumor patients before radiotherapy planning. While no significant association was found between [18F]F-FDG uptake and inflammation or bone loss, the study highlights the potential for PET/MR to support multidisciplinary decision-making beyond oncologic imaging [25].

## Discussion

This review aims to provide an updated overview from the past three years on the clinical impact of simultaneous [^18^F]F-FDG PET/MR in patients with HN tumors.

Recent studies focused on the advantages of PET/MR in detecting and delineating primary tumors, assessing lymph node status and metastatic spread, predicting aggressiveness and characterizing disease at a molecular level. PET/MR integrates the incomparable soft tissue contrast of MR with sensitivity of [^18^F]F-FDG PET, which is crucial for precise tumor delineation before radiotherapy or surgery. Accurate tumor volume estimation may avoid unnecessary treatments or complications.

Cao et al. reported that NPC typically spreads from proximal to distal regions; PET/MR may be able to prove that tumors often invade both sides of the nasopharynx, while bilateral invasion of adjacent regions is rare [4]. PET/MR could, therefore, be particularly beneficial for reevaluating and refining current CTV guidelines, which encompass bilateral skull base foramina irrespective of T classification [26] Terzidis et al. reported a 27.9% mismatch between the GTV_onco_ derived from PET/MR and combined with clinical information and the specimen-based GTV (GTV_patho_) in a small cohort of patients with tumors in the oral cavity, oropharynx and hypopharynx. GTV derived from PET alone (GTV_PET_) provided a more accurate agreement with the specimen-based GTV (GTV_patho_) compared to MR-derived GTV (GTV_MR_), but both PET and MR tended to overestimate the pathological tumor volume [13, 27, 28]. However, the findings require confirmation in a larger and more homogeneous cohort of patients. Nevertheless, comparison of tumor volumes based on in vivo multiparametric segmentation and segmentation of resected specimen remains a very challenging task, which also explains the paucity of published data regarding this topic. Although segmentation is done manually by radiation oncologists, it has inherent drawbacks depending on human expertise and suffering interobserver variability. Advances in deep learning for PET/MR segmentation might improve accuracy by standardizing tumor delineation [29, 30]. PET/MR may also influence clinical management by optimizing radiotherapy planning, helping to avoid unnecessary neck dissections, and supporting post-treatment surveillance. Its potential impact on multidisciplinary decision-making warrants further prospective evaluation.

PET/MR may outperform PET/CT in the evaluation of lymph node status, as suggested by Flygare et al. [11], more likely owing to the higher soft tissue contrast and fewer artifacts from dental hardware provided by MR than CT-counterpart. The capability of MR to detect factors such as skull base invasion, perineural spread, and extranodal spread (ENE) could enhance PET/MR's diagnostic accuracy, so influencing the therapeutic decision [31, 32]. Consequently, the experience of the observer in interpreting both MR and PET imaging can significantly impact the potential clinical benefit of using PET/MR in HNSCC patients.

The integration of PET and MR metrics may reflect imaging features associated with the aggressiveness of HNSCC in vivo. Meng et al. have developed a model combining SUV_mean_, K_trans_ and ADC values; the model has proven to distinguish poorly to moderately differentiated groups of HPSCC patients [8], and to outperform model considering PET or MR metrics separately.

The relationship between SUV values and ADC remains debated [12, 33]. A PET/MR composite model provides a comprehensive view of various tissue microstructure characteristics. DWI reflects the cellularity, proliferation rate and cell count; PET metrics could predict regional lymph node involvement and may reflect tumor biology related to disease aggressiveness, making prognostic assessments [34]. The comprehensive view of PET/MR may improve predictions of tumor characteristics and molecular expressions like EGFR and HPV status. In this regard, Chen et al. reported a combined model of SUV_mean_, SUV_max_, ADC_mean_, and perfusion parameters (Ve, Kep and K_trans_) for predicting the EGFR status with higher diagnostic utility than an uncombined model [15]. PET/MR may contribute to non-invasive prediction of EGFR status, potentially supporting patient selection in future studies. Similarly, a PET/MR model could assess microstructural tumor characteristics and predict the HPV status of the primary tumor. The non-redundant information of PET/MR provided in a single step could also effectively distinguish between CR and NCR to chemotherapy OPSCC patients [18]. In addition, the value of PET/MR could be further leveraged by examining its relationship with survival data. Our review found that PET metrics like SUL_peak_ and TLG, extracted from primary tumors, were reported as independent predictors of OS in HNSCC patients at multivariate analysis [19, 20]. This aspect could mean that OS may be more closely associated with the volume of the primary tumors, as well as with T and N stage, unlike cellularity. Furthermore, PET/MR incorporating functional MR sequences like IVIM and DCE-MR is associated with better survival predictions than traditional TNM staging in selected cohorts, by providing insights into tumor microenvironment characteristics, such as microvascular density (by DCE) and the blood circulation within randomly distributed capillaries (by IVIM), along with PET parameters [21, 35].

HNSCC patients could potentially benefit more from a simultaneous PET/MR scan than from separate scans, particularly for determining lymph node status before neck dissection. Crimì et al. demonstrated high accuracy with combined PET and MR models but cautioned that MR alone might lead to false positives if used solely for anatomic localization and attenuation correction [3]. In this study, SUV_max_ and SUV_mean_ cutoffs of 5.7 and 3.2, respectively, were suggested to enhance sensitivity. However, variability in hybrid scanners, different reconstruction algorithms and varying acquisition protocols can significantly affect their reproducibility.

The detection of occult lymph node metastases in clinically node-negative HNSCC patients remains a diagnostic challenge.

Cebeci et al. reported a relatively high sensitivity (83.3%) of PET/MR in the patient-based analysis in a cohort of 44 HNSCC patients with clinically negative necks [16]. This promising data should be confirmed by future studies, considering small cohort and the prevalence of pathologically proven N + (6/44 (13.6%)) and no level-based analysis.

Few PET/MR studies have focused on M staging or the detection of second-distant primary tumors. According to Katirtizidou et al., PET/MR and PET/CT exhibit similar performance for detecting malignant lesions located outside the HN [9]. Irrespective of performed analysis, e.g., per patient, per examination or lesion, the AUCs were above 0.944 for both PET/MR and PET/CT [9]. The similar performance could be attributed to the used contrast-enhanced fat-saturated sequences instead of DWI, for evaluating malignant lesions outside the HN region, such as the bone metastases, especially those with faint or absent [^18^F]F-FDG uptake [36]. [9]. The evaluation of disease outside the head and neck may involve characterizing lung nodules, where the resolution of CT may allow for a more accurate characterization than MR, although different opinions exist on this matter [37–39]. An older study reported the superiority of PET/MR over PET/CT in the management of oncologic patients, but noted potential discomfort due to the prolonged MR protocol used (mean, 66 min ± 12) [40]. Simultaneous PET/MR metrics appear more sensitive and specific for detecting the primary tumor, lymph node involvement or distant metastases in recurrent/metastatic NPC patients, compared with PET or MR metrics alone [41]. Furthermore, PET/MR seems to effectively predict post-treatment failure at both primary site and lymph node level in patients with sinonasal and NPC [23]. This could be particularly advantageous due to the critical nature of tumor sites included in this patient cohort, where the detection of perineural spread and/or skull base invasion is crucial for accurate image interpretation and subsequent selection of the optimal therapeutic strategy.

PET/MR appears to outperform PET/CT in the post-treatment setting, especially for tumor sites involving the oral cavity, nasopharynx or larynx. The tumor site plays a critical role in early detection of relapse. PET/MR may allow for a more accurate interpretation of findings, particularly in the nasopharynx and larynx, where the image interpretation with PET/CT may be affected by inflammation as well as physiological uptake in lymphoid tissue and muscles. Co-registered MR as opposed to co-registered CT could improve the differentiation of tumor and inflammation, because the signal intensity patterns of the two conditions differ from one another on T2-weighted sequences and DWI [30, 42]. However, the lack of well-designed prospective studies that head-to-head compare PET/MR and PET/CT for the evaluation of HN malignancies hampers to the possibility of drawing definitive conclusions about the best imaging modality in this setting.

Dental implants, inflammation, or local tissue alterations after radiotherapy can challenge diagnostic imaging. For example, dental implants cause more artifacts in PET/CT imaging [43]. Therefore, PET/MR seems to be the preferred modality for evaluating the oropharynx and the oral cavity, though affected by motion artifacts, like swallowing, talking, coughing, or breathing. Specific MR sequences capable of reducing these movements are often time-consuming, potentially affecting patient compliance during the scan. PET/MR may be preferable in patients with advanced disease, to solve unclear findings, such as postsurgical [^18^F]F-FDG uptake in the tongue, or assessing regions obscured by dental artifacts in CT [2, 43, 44]. Another potential advantage of PET/MR may be in the assessment of lesions with significant tumor necrosis, typically seen in more advanced stages of disease with weak or absent [^18^F]F-FDG uptake, unless superinfected.

Many retrospective studies, often with small cohort of patients, may hinder the drawing of definitive conclusions. The main issue is the relatively limited availability of PET/MR scanners, which requires considerable financial investment, both in the scanner and building infrastructure, affordable for large tertiary hospitals and/or academic centers. In addition to the economic burden, trained medical staff is essential to handle this modality. This ensures that PET/MR meets the clinical demands, avoiding redundant data. Apart from these factors, technical considerations such as different hardware properties, dedicated MR sequences for gamma signal attenuation correction, and varying reconstruction algorithms can considerably vary depending on the vendor of the scanner. This further limits the reproducibility of results obtained so far.

This review included only [^18^F]F-FDG studies, which are the standard PET radiopharmaceutical for oncologic diseases of the head and neck. This article focused on the clinical value of integrated PET/MR compared with current diagnostic tools, including PET/CT, rather than exploring new frontiers in nuclear medicine such as novel radiopharmaceuticals deemed useful in the head and neck region.

## Conclusion

Multiparametric and quantitative PET/MR may provide insight into the relationship between morphology, metabolism, cellularity, and perfusion in HN tumors. Several PET/MR-derived metabolic, diffusion, and perfusion parameters have shown associations with histologic grade, EGFR status, and outcome measures in preliminary studies. PET/MR has shown higher diagnostic performance than PET/CT for detecting locoregional HNSCC recurrence in selected studies. PET/MR is comparable to PET/CT for detecting distant metastases and second primary cancers in HNSCC patients. Larger prospective studies are needed to confirm these findings and define the appropriate MR-pulse sequences and the overall PET/MR acquisition protocol, which includes techniques for metal artifact reduction and motion correction to meet both clinical demands and patient compliance.
